# Non-Polar Lipids as Regulators of Membrane Properties in Archaeal Lipid Bilayer Mimics

**DOI:** 10.3390/ijms22116087

**Published:** 2021-06-04

**Authors:** Marta Salvador-Castell, Nicholas J. Brooks, Roland Winter, Judith Peters, Philippe M. Oger

**Affiliations:** 1 University Lyon, INSA Lyon, CNRS, UMR 5240, CEDEX, F-69621 Villeurbanne, France; marta.salvador-castell@insa-lyon.fr; 2 Department of Chemistry, Imperial College London, London SW7 2AZ, UK; n.brooks@imperial.ac.uk; 3 Faculty of Chemistry and Chemical Biology, Technische Universität Dortmund, 44227 Dortmund, Germany; roland.winter@tu-dortmund.de; 4 Université Grenoble Alpes, LiPhy, CEDEX, F-38044 Grenoble, France; peters@ill.eu; 5 Institut Laue Langevin, F-38000 Grenoble, France

**Keywords:** polyisoprenoids, squalane, archaea, cell membrane, permeability, fluorescence

## Abstract

The modification of archaeal lipid bilayer properties by the insertion of apolar molecules in the lipid bilayer midplane has been proposed to support cell membrane adaptation to extreme environmental conditions of temperature and hydrostatic pressure. In this work, we characterize the insertion effects of the apolar polyisoprenoid squalane on the permeability and fluidity of archaeal model membrane bilayers, composed of lipid analogues. We have monitored large molecule and proton permeability and Laurdan generalized polarization from lipid vesicles as a function of temperature and hydrostatic pressure. Even at low concentration, squalane (1 mol%) is able to enhance solute permeation by increasing membrane fluidity, but at the same time, to decrease proton permeability of the lipid bilayer. The squalane physicochemical impact on membrane properties are congruent with a possible role of apolar intercalants on the adaptation of Archaea to extreme conditions. In addition, such intercalant might be used to cheaply create or modify chemically resistant liposomes (archeaosomes) for drug delivery.

## 1. Introduction

Regardless of environmental constraints (pH, temperature, salinity, etc.), Archaea are found to inhabit the most extreme environments on Earth. These organisms are adapted to require severe environmental conditions that otherwise would have fatal effects. For instance, thermal fluctuations increase the disorder in phospholipid membranes and induce a phase transition to a higher disordered lipid phase [1]. In contrast, high hydrostatic pressures increase the ordering of lipids in the membrane and decrease the fluidity of the lipid hydrocarbon chains [2,3]. Such findings resemble the corresponding state principle for proteins [4] and might indicate that to yield functional organisms, the cell components of both non-extremophiles and extremophiles should harbor similar physicochemical parameters under their optimal growth conditions. Hence, a lipid membrane from a hyperthermophile microorganism, such as *T. barophilus*, which grows optimally at 85 °C, should present a fluidity and permeability at high temperature (ca. 85 °C) similar to that of the membrane of a mesophilic microorganism like *Escherichia coli* which grows optimally at 37 °C as stipulated by the homeoviscous adaptation theory.

**Figure 1:**
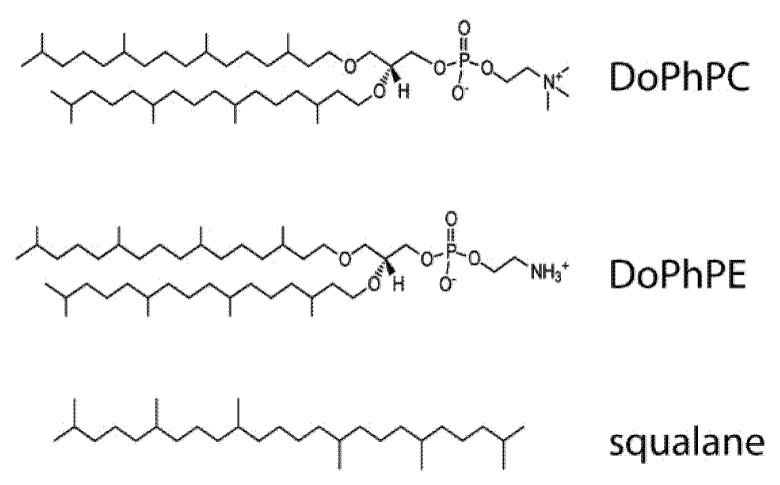
Skeletal formula of the archaeal lipids 1,2-di-*O*-phytanyl-sn-glycero-3-phosphocholine (DoPhPC), 1,2-di-*O*-phytanyl-sn-glycero-3-phosphoethanolamine (DoPhPE) and 2,6,10,15,19,23-Hexamethyl-tetracosane (squalane).

Archaeal membranes are composed of phospholipids with unique structures to keep plasma barriers functional under their habitual extreme living conditions. The structure of archaeal lipids is based on phospholipids with condensed isoprenoid hydrocarbon chains via ether bonds to an sn-2,3-glycerol phosphate backbone 5 (Figure 1). Furthermore, archaea may contain lipids with two polar heads, which can form a lipid monolayer [5,6] and are much more rigid and tightly packed [7,8,9], in contrast with the conventional lipid bilayer. The exceptional features of archaeal lipids have promoted research on such membranes. It is well-established, for example, that the presence of ether bonds, phytanyl chains and bipolar lipids confer unusually high stability to the archaeal membranes and make them highly impermeable to solutes [10,11,12,13,14].

Interestingly, apolar polyisoprenoids like squalene or its completely saturated form squalane, have been associated to the membranes as the most probable membrane regulators of archaea and particularly to those archaea adapted to live under extreme conditions [15,16,17]. Apolar polyisoprenoids are apolar molecules composed by several isoprenoid units (Figure 1) which have been localized in the bilayer midplane, perpendicular to lipids [18,19]. Such position may have an important impact on lipid bilayer properties. Until now, it has been demonstrated that the presence of apolar polyisoprenoids increases the membrane thickness and induces a membrane negative curvature [20]. Furthermore, it has been shown that lipid monolayers are also strongly influenced by the presence of apolar polyisoprenoids [21]. The main objective of this research is (1) to determine how the apolar polyisoprenoid squalane modifies the fluidity and the permeability to water and protons of an archaeal lipid bilayer mimic and (2) to determine to what extent apolar polyisoprenoids act as membrane regulators in archaea changing their physicochemical properties. The ability to change the lipid bilayer properties by adding apolar polyisoprenoids might be useful to create liposomes with functional characteristics similar to those of archaeosomes made with archaeal tetraether phospholipids. Indeed, the total synthesis of tetraether lipids is tedious, timely and cost ineffective due to extremely low yields, while the synthesis of diether lipids from glycerol-derivatives would be far more cost effective and would allow us to envision archaeosomes as an economically viable drug delivery tool.

Thus, we studied the impact of squalane on the permeability and fluidity of an archaeal lipid bilayer mimic composed of 1,2-di-*O*-phytanyl-sn-glycero-3-phosphocholine (DoPhPC) and 1,2-di-*O*-phytanyl-sn-glycero-3-phosphoethanolamine (DoPhPE) under high temperatures and high hydrostatic pressures (Figure 1). We present results of permeability studies to water and protons taking advantage of the carboxyfluorescein fluorescence (CF) self-quenching and pyranine fluorescent sensitivity to protons, respectively, and membrane hydration by generalized polarization of the fluorescent dye Laurdan. All of these support the role of squalane as an effective membrane regulator, able to create stable and impermeable diether-based archaeosomes. 

## 2. Results and Discussion

### 2.1. Solute Permeability

**Figure 2:**
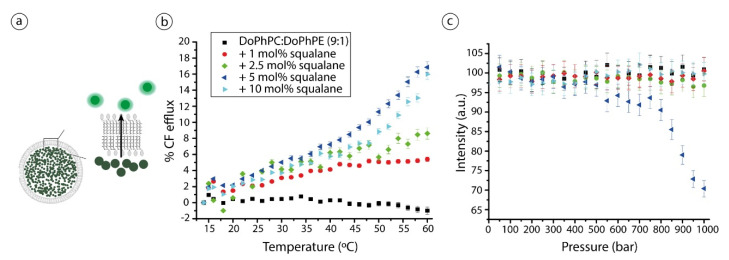
(**a**) Schematic representation of the CF efflux method, inside liposomes. CF is self-quenched but the fluorescence intensity emerges once it is released from liposomes due to a concentration decrease. The size of probes is not at scale. (**b**) The % CF efflux as a function of temperature and (**c**) CF normalized intensity versus high hydrostatic pressure applied on liposomes composed of DoPhPC:DoPhPE (9:1) in absence (black squares) or in presence of different squalane percentages: 1 mol% (red spheres), 2.5 mol% (green diamonds), 5 mol% (blue left triangles) and 10 mol% (cyan left triangles). Data represent triplicate (temperature) or duplicate (high hydrostatic pressure) measurements. No data points were excluded from the analyses.

CF efflux is a standard method to estimate the flux of large, uncharged molecules through lipid layers (Figure 2a). It is used as a proxy for water permeability. CF is encapsulated into liposomes at a concentration high enough to completely self-quench it and thus it presents a near zero fluorescent intensity [22]. Consequently, a CF efflux from the vesicles, as a result of external constraints such as temperature or pressure, will result in a net increase of fluorescent intensity, which can thus be monitored. As shown in Figure 2a (black squares), there is no release of fluorescence from liposomes made of DoPhPC:DoPhPE (9:1) as a function of temperature, meaning that they harbor a temperature-stable, extremely solute-impermeable bilayer. Previous studies by Nagle and colleagues have already shown that diphytanoylphosphatidylcholine (DPhPC), the ester-analogue of DoPhPC, presented a particularly low water permeability compared to their analogous acyl chains lipids, which has been attributed to the low local diffusion of water through isoprenoid ramified chains [23]. CF efflux is increased up to 4% at 60 °C when 1 mol% squalane is added. Hence, the permeability of these archaeal lipid bilayer model is enhanced by adding as little as 1 mol% squalane to the lipid mixture. Such effect is squalane-concentration dependent as the CF efflux increased up to ca. 17% at 60 °C at the higher concentrations of squalane. Permeability increased almost linearly with temperature at higher squalane proportions (Figure 2b). Since membrane permeability is highly correlated to membrane fluidity [24], the increase of CF efflux is a strong indication that squalane fluidizes the lipid bilayer. Incredibly low concentrations of squalane are able to modify the solute membrane permeability of the branched, archaeal-like lipids. The capability of changing the solute membrane permeability is well-demonstrated for cholesterol, the membrane regulator of mammalian cells [25,26].

Interestingly, the highest CF efflux is observed for the sample containing 5 mol% squalane and not the one with the highest quantity of squalane, i.e., 10 mol%. We have shown previously that the presence of squalane causes a lipid partitioning on DoPhPC:DoPhPE (9:1) between coexisting lamellar phases of different lipid, and most probably squalane, compositions [19]. The presence of such lipid phase domain borders promotes the creation of membrane defects that increase solute permeability [27,28]. Since squalane further promotes lipid partitioning in the membrane and lateral organization, the tendency observed for CF leakage might be explained by a difference in the number of membrane phase boundaries, due to a partitioning of squalane-rich and non-rich domains which could exist at 5 mol% squalane, and which would induce a higher CF efflux via the channel mechanism. On the other side, 10 mol% is at the maximal concentration of squalane that the membrane can accept, and such partitioning between squalane-rich and -poor domains may not occur, explaining why CF leakage is then reduced at this squalane concentration.

As expected, CF efflux is more pronounced at high temperatures (Figure 2b) but conversely, no CF efflux was detected from liposomes against high hydrostatic pressure (HHP) (Figure 2c), except in the case of the sample with 5 mol% squalane: its fluorescent intensity decreases at pressures above 500 bar which is somewhat unexpected. In fact, CF never presents a complete self-quenching in liposomes [22], essentially due to lipid-probe interactions, which under the increasing hydrostatic pressure conditions used here may allow the increase of the CF self-quenching (e.g., the decrease of the normalized intensity) if, for example, the volume of liposomes decreases. For instance, the fluorescent dye sulforhodamine at the same concentration is self-quenched at 99.9% in solution but only at 90% when encapsulated in liposomes [29]. In addition, it is well known that an increase in pressure can induce lipid and membrane domain segregation [30], causing a higher membrane tension and, once a critical domain size is reached, forming a bud to release such tension [31,32]. Budding of lipid vesicles are characteristic of phase-separated membranes and on multicomponent membranes, such as the one studied here. Budding has been observed in response to HHP [33], osmotic pressure [34,35] and the presence of enzymes [36] or detergents [37]. Such phenomena occur without pore formation in the bilayer and thus would not cause a CF release. As a direct consequence the overall volume of vesicles will decrease, and this may increase the probe concentration and consequently its fluorescence intensity and give a possible explanation for the observed CF intensity decreases for the sample with 5 mol% squalane. Furthermore, budding sites should be regions of high curvature, which is in accordance with previous SAXS results on the same lipid system which showed the tendency of squalane to induce the formation of non-lamellar phases in this lipid system [20].

### 2.2. Proton Permeabilty

**Figure 3:**
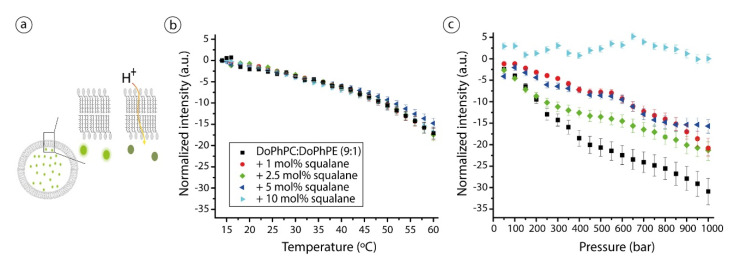
(**a**) Schematic representation of the pyranine technique to measure pH inside vesicles. Initially, pyranine is encapsulated in the liposomes and the entrance of protons will cause a decrease on pyranine fluorescent intensity. The size of probes is not at scale. (**b**) Pyranine normalized intensity inside vesicles as a function of temperature and (**c**) hydrostatic pressure applied on liposomes of DoPhPC:DoPhPE (9:1) in absence (black squares) or in presence of diverse squalane percentages: 1 mol% (red spheres), 2.5 mol% (green diamonds), 5 mol% (blue left triangles) and 10 mol% (cyan left triangles). Data represent triplicate (temperature) or duplicate (high hydrostatic pressure) measurements. No data points were excluded from the analyses.

Pyranine is a pH-sensitive dye which fluorescence has long been used to determine the pH inside phospholipid vesicles. After adding HCl outside of the vesicles, proton permeability of the lipid membrane is initiated. The flux of proton is determined over temperature and pressure scans via the monitoring of the pyranine fluorescence intensity (Figure 3a). It is important to keep in mind that there is no correlation between water and proton permeability in lipid membranes. All models presented to explain solute permeability fail when referring to proton permeation. Three molecular models have been proposed for proton permeation: the “defect” mechanism, the “water wire” mechanism and the “water cluster” model. Proton permeability is exponentially inversely proportional to the membrane thickness [24,38], but correlates weakly with fluidity while itself is highly correlated with water permeability suggesting different mechanisms for water and proton permeabilities. A fine example is the effect of cholesterol on the liquid ordered phase of bacterial lipids; it decreases water permeability but increases proton permeation. For detailed reviews, see [39,40,41].

Our results show almost no difference in proton permeability as a function of temperature for the archaeal membrane mimic in the absence or in the presence of squalane. We can observe for all samples a slight decrease of the pyranine normalized intensity, which indicates that for all samples the proton permeability increased slightly with temperature (Figure 3b). This increase in proton permeability is further accelerated for temperatures higher than 45 °C, with the exception of the sample with 5 mol% squalane which presented a proton permeability slightly lower than all other samples. Under HHP the DoPhPC:DoPhPE (9:1) mixture is quite permeable to protons, as seen from the much stronger decrease in pyranine fluorescence intensity. The presence of squalane in the lipid mixture strongly influences the lipid bilayer proton permeability (Figure 3c), although the decrease in proton permeability is not directly proportional to squalane concentration. However, at the highest concentration of 10 mol%, squalane completely blocked the decrease of the pyranine fluorescence intensity, and thus totally blocked proton transit inside the vesicles.

HHP is known to impact lipid molecular packing by modifying line tension and bending rigidity in liposomes [33,42]. Moreover, HHP can induce the partitioning of lipids, the formation of membrane domains or the merging of highly ordered domains [43]. All such membrane disturbances could easily result in the high proton permeation observed for liposomes composed of the DoPhPC/DoPhPE mix in absence of squalane when hydrostatic pressure is applied. In contrast, the presence of squalane in the membrane is expected to interfere with the proton transit mechanism. Protons need a traversal pathway to pass the highly hydrophobic region of the bilayer and travel through it. The presence of the apolar molecule in the bilayer midplane physically separates the lipid leaflets and therefore is expected to interrupt the proton pathway. Moreover, such phenomenon is congruent with our observation of a higher hydrocarbon core thickness in the presence of squalane [19,38]. At 10 mol% squalane concentration, this thickness is the highest [19]. All of these illustrates that the presence of squalane in the bilayer midplane prevents protons from passing between layers.

### 2.3. Lipid Bilayer Fluidity

**Figure 4:**
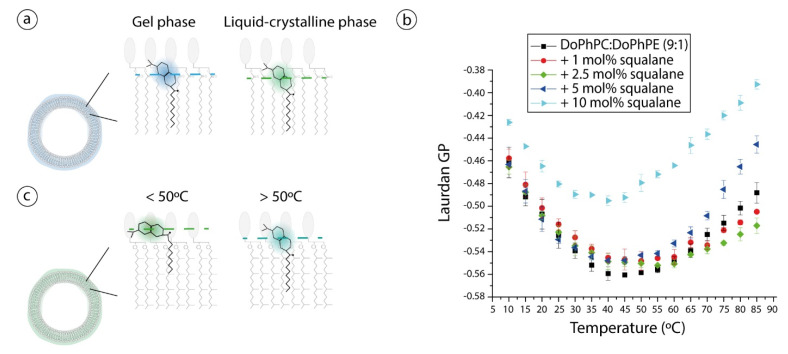
(**a**) Schematic representation of Laurdan position in a lipid bilayer composed by conventional diester phospholipids. While lipids are highly ordered in a gel phase, Laurdan emits in the blue region. A phase transition to a more disordered phase, the liquid-crystalline phase, induces an emission shift to green values. (**b**) Laurdan GP values from liposomes of DoPhPC:DoPhPE (9:1) in absence (black squares) or in presence of different squalane percentages: 1 mol% (red spheres), 2.5 mol% (green diamonds), 5 mol% (blue left triangles) and 10 mol% (cyan left triangles). (**c**) Schematic representation of Laurdan emplacement suggested for bilayers composed of archaeal-like diether lipids: Up to 50 °C, the fluorescent dye may adopt an “L-shape” form which would be consistent with the low Laurdan GP values. However, above 50 °C, there is a probe relocation to a deeper position that gives a change on dye emission and an increase on Laurdan GP values. Data represent triplicate measurements. No data points were excluded from the analyses. Dashed lines on (**a**,**c**) sketches represent Laurdan depth in the lipid layer.

Laurdan is a fluorescent probe sensitive to the polarity of its environment, and thus to its proximity to water molecules, and hence, its fluorescence depends on lipid packing and lipid phases [44]. When the probe is placed in a low polarity or high polar environment, it presents a blue or green emission, respectively. Consequently, the value of its general polarization (GP) informs about polarity: on the one side, in a fatty-acid based lipid bilayer Laurdan GP about 0.6–0.8 indicates that the phospholipid bilayer is under a gel phase and on the other side, values about (−0.4)–(−0.2) illustrate a more fluid state of the membrane, the liquid crystalline phase [45,46] (Figure 4a).

In the lipid systems studied here, up to 40 °C, GP decreases when increasing temperature, then, between 40 °C and 50 °C, it reaches a plateau and finally, above 50 °C, GP increases proportionally to temperature (Figure 4b). Remarkably, GP values are below −0.40 at all temperatures and in all samples, i.e., in absence and in presence of squalane. Such negative values are indicative of a liquid crystalline phase in membranes of familiar diester lipids. However, these extremely low values are not in agreement with the low water permeation and the slow motions attributed to archaeal diether lipids [14]. Moreover, the Laurdan GP increase above 50 °C is somewhat contradictory to the fact that temperature increases molecular motions, disorder and consequently water permeation and thus, GP should present lower values at higher temperatures [47,48]. The probe position in the lipid bilayer could explain the unusual Laurdan GP values. It was shown that, due to the highly rigid and tight membrane, Laurdan adopts an “L-shape” when anchored in tetraether monolayers [49]: the Laurdan’s chromophore group, i.e., the naphthalene rings, resides in the head group region perpendicular to lipids and only its hydrocarbon tail penetrates parallel to lipids in the hydrophobic region. Accordingly, contrary to the probe disposition when anchored in usual diester lipids, where the chromophore group is at 10 Å from the bilayer midplane [50], the naphthalene rings are highly exposed to water molecules in tetraether monolayers, which could explain the low GP values. A similar effect could account for the unusual negative GP values presented here, although in this case the membrane is a bilayer composed by diether phytanoyl lipids. We thus suggest that the phytanyl chains prevent the anchorage of the chromophore group inside the bilayer and thus it is highly exposed to water molecules in a similar way that on the “L-shape” disposition (Figure 4c).

The overall bowl shape of the Laurdan GP curve observed here is reminiscent of the gradual replacement of two coexisting phases of very similar physicochemical characteristics. In this context, the blue-shift emission observed at high temperatures could be explained by a probe relocation maybe due to a slow phase transition. An increase in temperature causes higher disorder in lipid bilayers, especially in their hydrocarbon chains, and it could lead to a deeper insertion of naphthalene rings into lipid bilayers at temperatures above 50 °C. This has been suggested to occur for Prodan, a probe similar to Laurdan, when it is placed in a more hydrophobic environment at high hydrostatic pressures [51]. Therefore, the effect of environmental changes in probe location needs to be considered. Interestingly, the sample with highest squalane proportion is the one that presents the overall highest GP values and in which the GP increase begins at the lowest temperature. Considering all previous interpretations, it would denote that the sample containing 10 mol% squalane presents a higher global fluidity since Laurdan is capable of being inserted more deeply in the lipid bilayer and thus presents higher GP values. Such fluidizing effect of squalene has recently been observed on asolectin liposomes [52].

## 3. Conclusions

The presence of squalane or similar isoprenoid molecules in archaeal membranes suggests that these apolar molecules may play a role in cell membrane adaptation to extreme conditions. Here, we have used archaeal phospholipid analogues to study the effect of squalane on permeation and hydration of archaeal bilayers at high temperatures and HHP. Laurdan GP demonstrates that apolar molecules, such as squalane, are capable of increasing membrane fluidity and, although without disturbing it excessively, it induces solute permeability through the lipid bilayer even at the lowest concentration studied, i.e., 1 mol%. However, probably due to its position in the midplane of the bilayer and an increase in bilayer thickness, squalane reduces proton bilayer permeability at high pressures, which could be essential for piezophile microorganisms. Interestingly, we obtained one more evidence that protons follow a different permeation pathway than other solutes through lipid bilayers, although this time in bilayers composed by archaeal-like lipids. 

Accordingly, it is noteworthy that squalane and, by extrapolation, other apolar polyisoprenoids, can modulate the physicochemical properties of the archaeal bilayer in a concentration-dependent manner. Since archaeal lipids are highly impermeable to solutes even in extreme conditions, the incorporation of polyisoprenoids would be an easy way to modify this essential characteristic. This would allow archaeal membranes to harbor similar permeability, fluidity, etc., in their optimal environmental conditions as non-extremophile cells, and also allow them to regulate their solute permeability depending on cell requirements. Last, the decrease in proton permeability facilitates the proton membrane gradient at extreme conditions and therefore basic functions, such as ATP production. All these facts indicate that polyisoprenoids may indeed play a role as a membrane regulator, a function comparable to that of sterols and hopanoids in eukaryal and bacterial cells, respectively. In future studies, it might be interesting to exploit these properties to create archaeosomes composed of only diether lipids, which may exhibit chemical and physical tolerance similar to those of tetraether-based archaeosomes at a fraction of the cost.

## 4. Materials and Methods

### 4.1. Materials

1,2-di-*O*-phytanyl-sn-glycero-3-phosphocholine (DoPhPC) and 1,2-di-*O*-phytanyl-sn-glycero-3-phosphoethanolamine (DoPhPE) were bought from Avanti Polar Lipids Inc. (Albaster, AL, USA). Purity guaranteed was >99%. 2,6,10,15,19,23-Hexamethyltetracosane (squalane), 5(6)-Carboxyfluorescein (CF), 8-Hydroxypyrene-1,3,6-trisulfonic acid trisodium salt (pyranine) and 6-Dodecanoyl-*N*,*N*-dimethyl-2-naphthylamine (Laurdan) were bought from Sigma-Aldrich Co (St. Louis, MO, USA).

### 4.2. Liposome Formation

DoPhPC and DoPhPE in a proportion of 9:1 were dissolved in a chloroform:methanol (2:1) solution. Squalane was dissolved in chloroform and added at different proportions to obtain 1 mol%, 2.5 mol%, 5 mol% and 10 mol%, respectively. Such solutions were vortexed, dried under a steam of nitrogen gas and left overnight under high vacuum to complete evaporation. Then, the lipid film was rehydrated with a given buffer, followed by vortexing, sonication for five minutes and five cycles of freezing/thawing. To form large unilamellar vesicles (LUVs), the solution was passed through a 100 nm polycarbonate filter by pressure extrusion 11 times at 55 °C using a Mini Extruder^®^ from Avanti Polar Lipids Inc. (Albaster, AL, USA). Consecutively, it was cooled down and the free dye was removed by chromatography over a prepacked P-10 desalting column from GE Healthcare. Liposomes were kept in ice and used immediately.

### 4.3. CF Efflux

When CF is trapped inside liposomes at high concentrations, i.e., >1 mM, it is mostly self-quenched, thus non-fluorescent [20]. Its leakage through the lipid bilayer will diminish its concentration and hence, its fluorescent intensity will increase. The buffer used was HEPES 10 mM, KCl 100 mM, EDTA 1 mM and CF 40 mM at pH 7.8. The final DoPhPC:DoPhPE concentration was 6 mM. Regarding the temperature measurements, the fluorimeter Jasco Spectrofluorometer FP-8500 was connected to a water circuit. For pressure measurements, we used a special fluorimeter equipped with a home-made chamber to create high hydrostatic pressures. The fluorescent excitation was placed at 492 nm and emission maximum was read at 518 nm. The permeability of CF was investigated in a temperature range from 10 °C to 60 °C and in a pressure range applied from 0 bar to 1000 bar. At the end of the temperature measurement, all trapped fluorescent dye was released by adding 0.1% Triton X100. Finally, CF efflux was calculated as [53]:

(1) $$\%CF\;efflux=\frac{F_{t}-F_{0}}{F_{max}-F_{0}}$$

where *F_t_*, *F*_0_ and *F_max_* are the fluorescence intensities at time *t*, time zero, and after total solubilization by Triton X100.

### 4.4. Pyranine Fluorescence

Pyranine is a pH-sensitive dye, this means that a pH variation causes a shift on its emission spectrum [54]. It has been largely used to detect pH changes inside liposomes [55,56,57,58]. In this fluorescent experiment, 5 mM HEPES at pH 7.5 together with 5 mM pyranine were used to rehydrate the lipid film. The final lipid concentration was 6 mM and LUVs were used immediately after size exclusion chromatography. pH permeability of lipid bilayer was investigated in temperature range from 10 °C to 60 °C and in a pressure range from 0 bar to 1000 bar. The first pH permeability measurement, called blank, was realized just by increasing the temperature or the pressure. Then, the measurement was repeated but this time adding 10 µL of HCl 0.1 M giving an initial pH 3 outside the liposomes. To separate the effect of temperature and pressure from proton permeability, we subtracted the blank to the second measurement. The fluorimeters used were the same as described in the CF efflux section. The excitation wavelength was 470 nm and emission was read between 500 nm and 520 nm with its maximum at 510 nm.

### 4.5. Laurdan Generalized Polarization

Laurdan is an environment-sensitive fluorophore widely used to quantify the lipid packing. The polycyclic aromatic compound naphthalene possesses a dipole moment that causes reorientation of water molecules which results in a red-shift of the probe emission [59]. Accordingly, after excitation at 350 nm, the emission spectrum of Laurdan in phospholipid membranes presents two local maxima, one at about 440 nm for ordered lipid (gel) phases and the other around 490 nm for the more disordered lipid phases (liquid-crystalline). 0.2 mol% of Laurdan was added to the lipids dissolved in chloroform/methanol. The lipid film was resuspended in HEPES 5 mM at pH 7.5 with a final concentration of 1 mM. To precisely quantify the polarity change, we used the general polarization (GP) term [44,45]:

(2) $$GP=\frac{I_{440}-I_{490}}{I_{440}+I_{490}}$$

where *I*_440_ and *I*_490_ are the emission intensities at 440 and 490 nm, respectively. GP values can range from +1 to −1, i.e., −1 being the highest lipid membrane fluidity.

The temperature-dependent measurements were performed on a K2 multifrequency phase and modulation fluorimeter (ISS Inc. (Champaign, IL, USA)). We used a quartz cuvette with a volume of 100 μL in a temperature range of 5–90 °C. Temperature control was achieved using a circulating water bath with an accuracy of ±0.1 °C.

## Data Availability

Not applicable.
